# Stroke Volume Monitoring: Novel Continuous Wave Doppler Parameters, Algorithms and Advanced Noninvasive Haemodynamic Concepts 

**DOI:** 10.1007/s40140-017-0235-4

**Published:** 2017-11-13

**Authors:** R. A. Phillips, B. E. Smith, V. M. Madigan

**Affiliations:** 1 Ultrasound and Cardiovascular Monitoring, Critical Care Research Group, School of Medicine, The University of Queensland, Brisbane, Australia; 2 Discipline of Intensive Care, University of Notre Dame Australia, Sydney, Australia; 3 Department of Anaesthetics and Intensive Care, Bathurst Base Hospital, Bathurst, NSW Australia; 4 University of Notre Dame Australia, Sydney, Australia

**Keywords:** Stroke volume, Continuous wave Doppler, Haemodynamic monitoring, Algorithms, Concepts

## Abstract

**Purpose of Review:**

Adequate oxygen delivery is essential for life, with hypoxia resulting in dysfunction, and ultimately death, of the cells, organs and organism. Blood flow delivers the oxygen bound in the blood, while haemodynamics is the science of blood flow. Stroke volume (SV) is the fundamental unit of blood flow, and reflects the interdependent performance of the heart, the vessels and the autonomic nervous system. However, haemodynamic management remains generally poor and predominantly guided by simple blood pressure observations alone.

**Recent Findings:**

Doppler ultrasound measures SV with unequalled clinical precision when operated by trained personnel. Combining SV with BP measurements allows calculation of flow-pressure based measures which better reflect cardiovascular performance and allows personalised physiologic and pathophysiologic modelling consistent with Frank’s and Starling’s observations.

**Summary:**

Doppler SV monitoring and novel flow-pressure parameters may improve our understanding of the cardiovascular system and lead to improved diagnosis and therapy. This review examines the physics and practice of Doppler SV monitoring and its application in advanced haemodynamics.

## Introduction

Stroke volume (SV), cardiac output (CO) and blood pressure (BP) are fundamental and independent measures of cardiovascular function, and are essential for the accurate understanding of cardiovascular (CV) physiology, pathophysiology and the guidance of fluid, inotropes and vasoactive therapies [1]. However, in clinical practice, BP and BP-derived surrogates of SV are used interchangeably in place of direct and accurate flow measurements. This conflation of flow and pressure potentially compromises our understanding of CV physiology and limits the effectiveness of goal-directed therapy (GDT) and the clinical utility of haemodynamics [2, 3•, 4]. It may also result in inappropriate therapies, adverse outcomes and sub-optimal management [5–7, 8•].

The arterial system is a network of branches of changing diameter, with changing BP, flow and pressure waveforms throughout its distribution [9, 10]. Further, regional vascular tone changes beat-to-beat under the control of the autonomic nervous system (ANS), diminishing the value and reliability of peripheral BP measurements as an analogue of SV. Understanding what is actually being measured, both where and when, is vital when evaluating the circulation and monitoring the effect of therapy.

Guyton observed that BP was the product of CO and systemic vascular resistance (SVR) or ‘total peripheral resistance’ [1]:

$$ \boldsymbol{MAP}=\boldsymbol{CO}\times \boldsymbol{SVR} $$

However, as CO is the product of stroke volume and heart rate (HR), then:

$$ \boldsymbol{MAP}=\left(\boldsymbol{SV}\times \boldsymbol{HR}\right)\kern0.5em \times \boldsymbol{SVR} $$

Thus SV is the fundamental unit of CV function as regards blood flow delivered from the heart to the circulation, and represents the combined influences of the heart, blood vessels, the ANS and even the physical properties of the blood itself. The SV and SVR are interdependent and compensatory variables acting in concert, conducted by the ANS, to preserve BP and perfusion. Importantly, they are treated with distinct therapies: stroke volume predominantly with fluid and inotropes, and SVR with vasoactive agents.

It is also clear that BP and SV are distinct measures and cannot be used interchangeably. Substitution of SV with BP-derived estimates of SV is a physiological example of mathematical coupling which undermines advanced haemodynamic monitoring. An independent and accurate method of measuring SV is essential to complement the measurement of BP in order to understand the function of the circulation, as Frank, Starling and later Guyton, established [11–13].

Importantly, the role of fluid, inotropes and vasoactive interventions is directly targeted to change SV, so accurate and sensitive measurement of SV is central to diagnosis and therapy of the cardiovascular system.

While this circulatory overview is fundamental, the accurate measurement of SV has not previously been routinely available, making the effective implementation of basic haemodynamic monitoring impossible.

## Measurement of Stroke Volume

The accurate measurement of SV is central to understanding advanced haemodynamics and has become the Holy Grail of circulatory assessment. However, as SV, by definition, is the flow per beat through the ventriculo-arterial valves, a flow measurement technology must be used to measure flow across either the pulmonary or aortic valves (PV or AV). Historically, many technologies have been used to estimate SV, with most measuring flow surrogates at various points in the circulation from which an estimate of SV is made using a transfer function. The further the measures are made from the ventriculo-arterial valves, the greater the influence of the ANS and SVR variation, and the less agreement the method has with flow, and the less reliable the technology.

## Estimation of Stroke Volume from Oscillometry

Simple oscillometric measures of brachial BP have been ubiquitous since the pioneering work of Riva-Rocci in 1896 [14]. Although the most common circulatory measurement, oscillometry has significant sources of error leading to questionable reliability [15–20]. While measurement of the BP is routinely performed at the level of the brachial artery with an upper arm cuff, the choice of referencing systolic, diastolic or mean arterial pressure (MAP) further confuses applicability of the method. Regardless, oscillometric BP is a poor surrogate for SV as Guyton predicted and Wo and Shoemaker et al. demonstrated [1, 21].

## Estimation of Stroke Volume from CO and Dilution Methods

Several methods measure CO, the minute output of the ventricle, including the Fick method and partial CO_2_ rebreathing techniques, thermodilution with the pulmonary artery catheter (PAC) and other indicator dilution techniques. CO is then divided by HR to yield the average SV. From 1970 to 2010, the PAC was regarded as a reference standard, but recent evidence suggests that not only is its absolute accuracy questionable, it has limited sensitivity in terms of detecting changes in SV. Further, there is little evidence of outcomes benefit; it is associated with significant patient risk; it requires time to set up and perform, and has limited application to critically ill patients [22, 23•, 24–28]. While the Fick [29] method is considered accurate and reliable, it is not generally used in critical care patients, let alone the haemodynamically unstable patient seen in other clinical environments.

## Estimation of Stroke Volume from the Arterial Pulse Waveform

The use of pressure transducers to measure intra-arterial BP waveforms, with accompanying transfer functions to estimate SV, have also proven unreliable. This is particularly true in hyperdynamic circulations where these methods have limited value for guidance of interventions [30–34]. These methods, which include LiDCO (LiDCO, London, UK), PiCCO (Pulsion Medical Systems, Munich, Germany), Deltex CardioQ-ODM+ (Deltex, Chichester, UK) and Vigileo/Flotrac (Edwards LifeSciences, Irvine, CA, USA), are predicated on the assumption that the ANS is constant between and within individuals and over time, resulting in SVR being mathematically predictable. This flawed assumption results in insensitivity to acute SV change, despite most devices recommending intermittent calibration to a alternate monitoring technology such as thermodilution or oesophageal Doppler. Additionally, the algorithms used by different manufacturers are proprietary and not directly comparable, resulting in absolute output values of poor agreement and interchangability, with none as accurate as Doppler ultrasound [30–36]. Further, any predictability of the ANS functions, vascular tone and fluid dynamics breaks down in many common clinical situations. These include, but are not limited to, high, low and changing outputs as in sepsis and trauma, during dyssynchronous breathing, in dysrhythmias, with vasoactive interventions, in right ventricular overload states, peripheral vascular disease and in children, precisely the patients in whom the measurement of SV is critical [30]. This results from the mathematical coupling of BP and SV and is a fundamental source of error in haemodynamics. This limits the applicability of BP-derived haemodynamics and underlies the failure of many GDT protocols; guiding therapy by BP and a BP surrogate of SV can only be as effective as guiding it by BP alone [3•, 4, 5, 34–37].

Other devices use plethysmographic or pulse oximetric measurements in the digits (Masimo, Irvine, California, USA; Nihon Kohden, Tokyo, Japan; Edwards LifeSciences, Irvine, California, USA), and then estimate the central circulation. However, as the measure reflects blood flow characteristics in the distal digital circulation, the region most markedly affected by circulatory redistribution, it is unlikely to provide any more than the circulatory status of the digit and provide little reliable information on the central circulation.

## Estimation of Stroke Volume Using Bio-impedance

Similarly, non-invasive methods based on thoracic electrical impedance or reactance such as NiCOM (Cheetah Medical, Wilmington, Delaware, USA) have failed to demonstrate sufficient accuracy and sensitivity in critical care practice [37–45].

Unfortunately, with the exception of techniques based on the Fick principle, the reliability of all of these methods for measurement of SV has been found to be generally inadequate for monitoring and therapy in the critical care environment where haemodynamic variability is common and can be critical [27, 34–40].

## Measurement of Stroke Volume Using Doppler Ultrasound

Doppler ultrasound as used in echocardiography is a reliable method for measuring flow volumes, providing validated direct transvalvular measurement of SV and CO [36, 46, 47, 48••, 49–54]. The ultrasonic cardiac output monitor (USCOM 1A, Uscom Ltd., Sydney, Australia) is a specialised Doppler SV monitor derived from echocardiography, which has similarly been validated for CO ranging from 0.12 to 18.3 L/min [55–56]. These non-invasive flow measurements can be integrated with BP measures to generate integrated flow-pressure parameters and monitor changes predicted by pressure-volume loops. This may lead to improved understanding of cardiovascular physiology and pathophysiology, and ultimately to physiologically guided therapy with improved outcomes in adults and children [2, 58, 59].

### Doppler Ultrasound Flow Measurement

The measurement of cardiac flows using Doppler ultrasound was first described by Franklin in 1961 [46]. By the early 1980s, non-invasive measurements of SV using Doppler ultrasound across both the aortic and pulmonary valves were validated against the reference methods for reliability, reproducibility and sensitivity [50–54].

The elegance of the Doppler method is that the SV is measured directly by a flow sensor at its precise point of delivery from the ventricles to the aorta or pulmonary artery, the only two points at which the SV can be directly measured. Conversely, the further from the ventriculo-arterial valve that the flow is measured, the less precise the estimation of SV becomes as other haemodynamic factors and the ANS intervene. Given the complexity of physiologic reguation and the coupling of the cardiac and vascular functions, transvalvular Doppler measurements are likely to become the true gold standard clinical measurement of SV. Doppler ultrasound is non-invasive, well validated, cost effective and relatively simple to implement in a wide range of subjects. In addition, its sensitivity to change of SV’s less than 5% makes its implementation in clinical practice compelling [26, 57–60].

### The Doppler Method

Doppler ultrasound accurately measures changes in velocity over time, which can be applied to the heart and vessels to detect blood flow haemodynamics. Once we know the velocity of flow through a vessel and the diameter of that vessel, then the SV and the CO can be calculated using the following equations [47, 48••, 60, 61].

If the frequency of the emitted beam is fe, and the sound frequency observed (i.e. reflected) is fo, then the Doppler equation states:

$$ \boldsymbol{fo}=\left(\frac{\boldsymbol{C}+\boldsymbol{Vr}}{\boldsymbol{C}+\boldsymbol{Vs}}\right)\boldsymbol{fe}\kern0.75em \boldsymbol{or}\kern1em \boldsymbol{fo}=\left(1-\frac{\boldsymbol{Vs}-\boldsymbol{Vr}}{\boldsymbol{C}}\right)\ \boldsymbol{fe} $$

where *C* is the velocity of sound in the medium (a constant and approximately 1570 m/s in blood and tissue), Vr is the velocity of the receiver relative to the medium and Vs is the velocity of the source relative to the medium.

The change in observed frequency resulting from relative motion of the source and the observer is therefore:

$$ \boldsymbol{\varDelta} \boldsymbol{fo}=-\left(\frac{\boldsymbol{Vs}-\boldsymbol{Vr}}{\boldsymbol{C}}\right)\ \boldsymbol{fe} $$

Red blood cells are efficient reflectors of ultrasound. If the observer, i.e. the ultrasound transducer is stationary, then any frequency change relates to the velocity of the red blood cells, assuming that the flow is aligned with the observing probe.

**Fig. 1:**
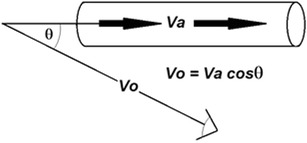
Relationship between blood flowing through an artery at actual velocity *Va* and an oblique ultrasound beam at angle demonstrating the observed velocity *o*, which is less than *Va* by a factor of cosine Ɵ

While the Doppler principle assumes insonation parallel to the line of flow, the Doppler method is relatively insensitive to errors related to oblique insonation as shown in Fig. 1.

If the angle between the observer (transducer) and the flow axis is 18°, then the cosine of the angle is 0.951 (Fig. 1). This means that the observed velocity Vo will be 95.1% of the actual velocity Va at 18°. Therefore, within a ± 18° sector (or 36° in total) along the line of flow, the measured velocity will be no less than 95% of the true value, well within clinically acceptable limits. Integrating the two equations and assuming the source (transducer) velocity is 0 yields the Doppler equation:

$$ \boldsymbol{\varDelta} \boldsymbol{fo}=2\boldsymbol{f}\ \boldsymbol{v}\ \frac{\boldsymbol{Cos}\varnothing }{\boldsymbol{c}} $$

## Conversion of Flow Velocity to Flow Volume

The Doppler equation defines the relationship between frequency and velocity, and from velocity, the flow volume can be calculated by multiplying by the cross-sectional area (CSA) of the vessel. For a valve of radius *r* with blood flowing at a velocity *V*, and with a circular CSA, then at steady-state, the quantity of fluid flowing per unit time (Qt) or flow volume is:

$$ \boldsymbol{Qt}=\boldsymbol{v}\kern0.5em \times \boldsymbol{\pi} {\boldsymbol{r}}^2 $$

**Fig. 2:**
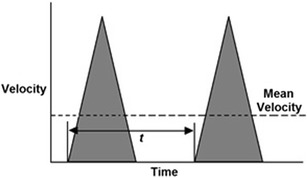
Ventriculo-arterial Doppler velocity-time flow profile, with the area of the triangle being the mean velocity or velocity-time integral (vti), and the time between cycles the time or 1/HR

As the blood flow is pulsatile, we need to know the mean velocity of the blood flowing across the aortic or pulmonary valve. The ejection waveform at the aortic valve approximates a triangle, with a normal velocity range of zero at the base to around 1.4 m/s at the peak as shown in Fig. 2. The duration of systole is approximately 350 ms, and diastole around 450 ms, giving a total cycle time of around 800 ms (HR = 75 bpm) denoted by *t*. The mean velocity of ejection can be calculated from the area under the ejection curve by integrating the velocity with respect to time *t*. This is the velocity-time integral or vti, and is known as the stroke distance (sd) as it is the average distance red blood cells travel per heart beat, normally around 25 cm.

SV is the product of the stroke distance, vti, and the cross-sectional area (CSA):

$$ \boldsymbol{SV}=\boldsymbol{vti}\times \boldsymbol{CSA} $$

where CSA = πr^2^. The ejection Doppler flow profile can be traced to calculate vti, but the radius of the ventriculo-arterial valve is required to calculate CSA and thereby, SV. There are several possible methods to obtain the CSA.

## Echocardiographic Measurement

**Fig. 3:**
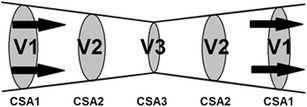
As the CSA narrows, the velocity of flow increases, as the flow volume of blood at any single point equals that at any other point, or CSA1 × V1 = CSA2 × V2 = CSA3 × V3 = Qt. The continuity equation dictates that the measurement of velocity must be made at exactly the same point as the measurement of CSA or the calculation of flow will be erroneous. Combining the velocity measured at CSA1 with the cross-sectional area at CSA3 would result in an erroneous measurement

The diameter of the aortic or pulmonary valve, or the left or right ventricular outflow tract (LVOT/RVOT) can be measured using echocardiography [62]. However, the value for velocity that is used to calculate vti must be measured at exactly the same point as the CSA of the flow to comply with the continuity equation. For example, where a vessel narrows from a maximum diameter and cross-sectional area CSA1 to a minimum cross-sectional area at CSA3 and then widens again to its original size (Fig. 3), then the continuity equation states that the product of the velocity and CSA (the flow) at any one point equals the product of the velocity and CSA at any other point. Thus, as the vessel narrows, the CSA diminishes but the velocity increases.

**Fig. 4:**
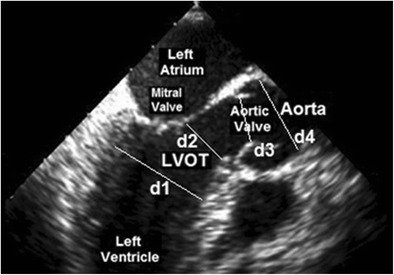
Apical 2 chamber view of the left ventricle demonstrating the LVOT, the aortic valve and the aorta, and the different diameters at each point d1 to d4

The LVOT varies in diameter across its course (Fig. 4) and the continuity equation determines that the flow volume across any of these points, d1, d2, d3 and d4, must be equal, and so the velocity must vary as in Fig. 3. Coupling the exact position for measuring both Doppler flow and CSA is essential to prevent violation of the continuity equation, but this is technically challenging in practice. Fortunately, the physics of continuous wave (CW) Doppler simplifies the method.

### Pulsed-Wave Doppler v Continuous-Wave Doppler

A CW Doppler transducer emits a continuous beam of ultrasound and continuously detects and measures the reflected echoes regardless of where they originate. The ultrasound beam diverges from the transducer face as a cone, resulting in a large volume of tissue being ‘scanned’ for signal frequency shift.

With pulsed wave (PW) Doppler, a timed pulse is emitted from the transducer and the transducer then ‘listens’ for the returning echo. As the speed of sound in fluid is a constant (approximately 1570m/s), the time delay allows determination of the depth of the reflected signal similar to detection of the depth of a submarine using sonar ‘pings’. Setting an exact time delay from the emission of the pulse to ‘listening’ for the echo creates a sample volume at a fixed distance from the transducer from which signals are measured. This sample volume represents the small and exclusive target volume from which the Doppler signals are measured [48••, 61].

While PW Doppler has specific applications, it has limitations when measuring SV. There is a maximum rate at which the ultrasound pulses can be emitted, reflected and received before the next pulse can be generated; the sample rate is consequently limited by the speed of sound in tissue and the depth of the target. This gives a finite limit, the Nyquist limit, to the pulse repetition frequency which is a function of the velocity of the target flow and the depth at which the sample volume is placed. High velocities and deep sample volumes may result in inaccurate velocity and volume measurements.

**Fig. 5:**
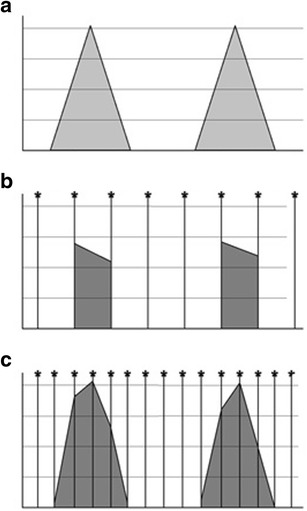
Effect of increasing sample rate and increasing accuracy of velocity flow profile reconstruction. The higher the sample rate the more accurate the waveform reconstructed. PW Doppler is limited by sample rates, while CW Doppler is not

In Fig. 5, the true velocity flow profile of aortic ejection is demonstrated in panel (a); panel (b) shows the apparent waveform as measured with a low frequency of 6 Hz, resulting in an inaccurate waveform, with inaccurate calculation of flow velocities and volumes. Doubling the frequency of sampling to 12 Hz as in panel (c) results in a much more valid representation of the true ejection waveform with consequent increase in accuracy of measurement of ejection velocity and volumes.

CW Doppler does not have this limitation, as it measures the highest velocity, which is at the level of the valve leaflets. Combining this Doppler signal with the minimum diameter (which is also at the valve leaflets) provides a precise method of flow and stroke volume calculation, in accordance with the continuity equation. PW Doppler is generally reserved for lower velocity flows at defined points such as the mitral inflow and right and left atrial venous inflows. Ihlen, Moulinier and others have confirmed superior signals and reduced inter-observer error using CW Doppler [52, 53]. Kusumoto reported a 2% variability of CW Doppler measures compared with 23% variability for PW Doppler measurements [54].

### Converting the Doppler Waveform to a Flow Volume Measurement

When using PW to measure flow volume, the sample volume should be placed 1 cm into the outflow tract, and the CSA measured at that same point as the continuity equation dictates [62]. This is not as straightforward as it might seem; the sample volume and measured CSA must be fixed in relation to the heart and the flow to ensure that the sample volume does not move relative to the outflow tract during the normal cardiac cycle. However, the heart moves in all three planes during systole and diastole and also with respiratory excursion. While the sample volume is fixed relative to the transducer and chest wall, the heart and the blood flow is moving across and through the PW sample volume. This means that the sample volume is ‘waved’ through the outflow tract thereby measuring velocity at differing points throughout the cardiac cycle, which violates the principle of the continuity equation and increases measurement error.

## Flow Cross Sectional Area Measurement and Limitations

**Fig. 6:**
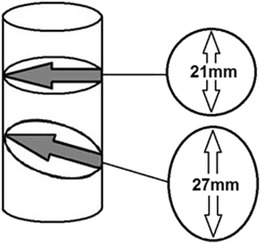
Effect of oblique transection of a cylinder using 2D ultrasonic measurement of flow diameter demonstrating that a 3-mm error in measurement of the flow radius r produces a 65% error in volume calculation when squared in the CSA = πr^2^ formula

Direct 2D ultrasonic measurement of the valve radius introduces potential errors secondary to oblique insonation and also from the spatial resolution of 2D ultrasound [60], both of which contribute significantly to errors in CSA calculation and thereby of SV. Mark et al. reported ‘2D measurement of aortic diameter was time consuming and poorly correlated with invasive measurements (r=0.31)’ [63]. Figure 6 illustrates the error resulting from measuring the flow diameter with an oblique ultrasound beam. The apparent CSA becomes oval with an increased measured diameter of the vessel. While the linear error of *r* may be small, once calculated as CSA = πr^2^, any error is squared, leading to a significant error in the calculated CSA. An error in radius measurement of 3 mm results in over-measurement of the CSA from 346 to 572 mm^2^, an error of 65%, which results in a flow volume measurement error of the same magnitude.

To ensure accuracy of the flow radius measurement, The American Society of Echocardiography (ASE) recommend averaging at least three measures of the flow diameter, and in dysrhythmia, 5 or more may be required [62]. While this improves the accuracy of echocardiography measurements, it increases the examination time considerably.

Measurement of the valve area using 2D ultrasound is further limited by the spatial resolution of the pulsed ultrasound beam. The frequency of the transducer *f* is inversely proportional to the wavelength *λ*, hence resolution increases directly with frequency:

$$ \boldsymbol{C}=\boldsymbol{f}\boldsymbol{\lambda } $$

where *C* = velocity of ultrasound, *f* = frequency of the ultrasound beam and *λ* = wavelength of the pulse [61]. If each 2D ultrasound pulse is between 2 and 4*λ*, the spatial pulse length (SPL) of a transducer where *f* = 5 MHz is 1.2 mm, and the spatial resolution is 0.6 mm, as 2D axial spatial resolution = SPL/2.

If 0.6 mm is the 2D axial spatial resolution, then it is also the minimum standard deviation of any set of measures, which makes the minimum 95% confidence interval 1.2 mm. If we apply that minimum error to the calculation of the CSA for a 2-cm diameter valve, then *r* is 10 ± 1.2 mm, and the minimal CSA error is ± 24%.

Errors in measurement of the CSA, whether from oblique insonation or poor 2D spatial resolution, are the biggest single source of inaccuracy in echocardiographic measurement of SV. If changes in SV of 15% or less are to be accurately measured during clinical therapy, then this measurement requires meticulous technique and repeated measures to achieve acceptable sensitivity.

### Morphometric Estimation of Valve Radius

From the above, clearly the highest velocity in the ventricular outflow tract will occur at the narrowest point, which is at the level of the aortic or pulmonary valve annulus at the point of leaflet insertion [50]. Anthropometry demonstrates that cardiac dimensions, including normal cardiac valves, show a linear relationship with body height and surface area, and can be determined with high accuracy using morphometric algorithms [64–69]. Nidorf et al. and Sheil et al. reported excellent correlations of body height with AV diameter (*r* = 0.96 and 0.93 respectively) [67, 68]. The morphometric method also has the advantage that body height is more readily and sensitively measured than valve radius using ultrasound, as discussed above. As the valve annulus is predominantly fibrous and forms part of the stiff cardiac skeleton, it is less likely to change significantly over time or during any clinical episode. Consequently, serial monitoring, using the morphometric method, is likely to provide a more reproducible estimate of valve radius, and therefore a more accurate absolute and serial measure of SV.

## Oxygen Delivery and Consumption

Once a reliable measure of SV has been acquired, combining this with other common physiologic measurements allows the calculation of a number of important parameters of cardiovascular and pulmonary performance.

## Oxygen Delivery and Consumption

Once the SV and CO are known, then combining these with arterial saturation SaO_2_ and haemoglobin concentration [Hb] allows calculation of the volume of oxygen delivered per minute, DO_2_. Dividing this by body surface area (BSA) yields oxygen delivery index (DO_2_I):

$$ {DO}_2=\frac{1.34\;x\;\left[ Hb\right]\;x\  CO\ x\ {SaO}_2}{100} $$

The Fick equation [29] states:

$$ CO=\frac{VO_2}{CaO_2-{CvO}_2} $$

where VO_2_ = minute consumption of oxygen, CaO_2_ = arterial oxygen content and CvO_2_ = venous oxygen content. If the venous and arterial oxygen contents are known, then it follows that VO_2_ is simply:

$$ {VO}_2=\left({CaO}_2-{CvO}_2\right)x\  CO $$

Again, this can be indexed to BSA as VO_2_I [70]. The oxygen extraction ratio is then simply the ratio of VO_2_ to DO_2_.

## Combined Flow-Pressure Indices

Flow and pressure are the fundamental physical parameters of the cardiovascular system. While much of our understanding of disease and treatment has derived solely from the measurement of BP, combined flow-pressure measurements allow the calculation of pressure-volume coupling and combined flow-pressure parameters [71–75].

With accurate measures of SV, direct measures of ventriculo-arterial coupling can be derived with input of BP values. For example MAP = CO x SVR [1].

$$ \mathrm{or}\  SVR=\frac{MAP}{CO} $$

However, if we assume that blood flow requires kinetic energy (KE) and BP represents potential energy (PE), then the product of the two energy components is the total stroke work (SW) of the heart in milli-Joules (mJ).

$$ SW=\frac{60}{450}x\  SV\ x\;\left( MAP- CVP\right) $$

Power is the rate of performance of work, so dividing SW by the time of one heart beat (60/HR) provides the work per unit time, while SW × HR is cardiac power output (CPO) for 1 min [76–80].

$$ CPO=\frac{\left( SV\;x\; HR\right)\;x\;\left( MAP- CVP\right)}{450.037} $$

Arterial elastance (Ea) is the ratio of pressure change to volume change in the pressure-volume loop, or dP/dV, and can be noninvasively calculated as pulse pressure (PP) divided by SVwhere PP = BP_systolic_ − BP_diastolic_, and SV = left ventricular end diastolic volume − left ventricular end systolic volume (LVEDV − LVESV) [81–84].

$$ \mathsf{Ea}=\mathsf{dP}/\mathsf{dV}=\mathsf{PP}/\mathsf{SV} $$

Arterial compliance (Ca) is the reciprocal of elastance or dV/dP and can be noninvasively calculated as SV/PP [82].

$$ \mathsf{Ca}=\mathsf{dV}/\mathsf{dP}=\mathsf{SV}/\mathsf{PP} $$

These combined flow-pressure parameters show increased predictive value for outcomes in heart failure, hypertension and cardiogenic shock [76–80], provide an improved understanding of the Frank-Starling mechanism [79] and improve guidance of fluid, inotropes and vasoactive interventions.

### Central Arterial Pressure

While oscillometric brachial BP remains a widely adopted clinical standard, central aortic BP measured using suprasystolic oscillometry correlates more closely with measures from aortic catheters in adults and children [85–87]. Evidence is accumulating that central BP measures are likely to provide more relevant physiologic values for flow-pressure derived parameters, as well as more appropriate therapeutic guidance in hypertension [88, 89].

### Inotropy

Inotropy, or myocardial contractility, is a familiar concept of cardiac performance*,* but seldom thought of as a quantitative clinical parameter [72–74]. Conceptually, a ‘weak’ or failing ventricle lacks power compared to a healthy heart which is ‘strong’. Inadequate inotropy, in the absence of ANS-mediated vasoconstriction, results in hypotension. Inadequate inotropy may characterise myocardial infarction, cardiomyopathy and other primary cardiac conditions, as well as reflecting myocardial depression secondary to anaesthesia, sepsis, pancreatitis, hypoxaemia, toxic and deranged biochemical states, amongst others, meaning that almost all acutely ill patients will have some degree of myocardial impairment.

Smith and Madigan developed a novel approach to measuring inotropy based on effective energy transfer from the ventricle to the aorta [90]. Using Doppler measured SV and BP, they calculated total SW, and divided this by the ventricular ejection flow time (FT), the duration of energy expenditure, to derive inotropy. While CPO has been measured previously using similar parameters and invasive measures, this has been calculated as an average over the duration of the entire cardiac cycle. The limitation of this ‘averaging’ method is that CPO is rate-dependent and assumes a fixed ratio of systole to diastole and is therefore limited at high and low heart rates and in dysrhythmias. In addition, CPO is usually calculated only from MAP and CO and therefore only measures the PE element of total cardiac work, ignoring the kinetic energy element. By indexing summated PE and KE to the exact duration of systole (FT), the instantaneous power output of the ventricle can be measured. As the heart obeys the ‘all or nothing rule’, this instantaneous power output must be a direct function of myocardial contractility or inotropy, and should be largely independent of loading conditions. To differentiate this from other measures of inotropy, this is known as the Smith-Madigan Inotropy (SMI), which when indexed to body surface area becomes the Smith-Madigan Inotropy Index (SMII). The product of the formula gives power or SMI in watts, the SI unit of power. For a typical adult, the value of SMI is around 3 to 5 W, and for SMII around 1.6 to 2.2 W/m^2^. [90].

### Potential to kinetic energy ratio – vascular impedance

By analogy to alternating current modelling in electronic circuits where impedance *Z* equals instantaneous voltage divided by instantaneous current, *Z* = *V*_inst_/*I*_inst_, the instantaneous impedance for the left ventricle is given by the instantaneous pressure in the aorta divided by the instantaneous flow in the aorta, which is directly proportional to the ratio of PE to KE. This is the PE to KE ratio or PKR, and is a measure of the balance which must exist between blood pressur*e* and blood flow in the circulation. One without the other is of no physiological value. In health, around 96% of SMI generates blood pressure while only 4% generates blood flow. This gives a PKR of around 25:1. Excessive vasodilation, as occurs in sepsis, anaphylaxis or following extensive neuraxial anaesthesia for example, leads to an excessively high flow but inadequate MAP. The net result is that PKR falls to values as low as 5:1. Low PKR values therefore indicate excessive vasodilation and circulatory collapse. High values of PKR on the other hand indicate high vascular impedance as found in vasogenic hypertension or with excessive vasopressor use. Consequently, not only does PKR quantify the degree of imbalance of the circulation, it also indicates the direction in which treatment should be targeted to return the circulatory balance to normal.

## Doppler Ultrasound Technologies

Currently, there are three Doppler-based methods in clinical practice.

### Echocardiography

While echo is the longest established method of measuring SV, it remains technically challenging. The American Society of Echocardiography in its position paper on monitoring conclude that echo is highly user dependent, requires at least 2 years of full time training and that there is minimal outcomes evidence of its effectiveness as a monitor [91•]. Because of the time per examination, 30–60 min, the 2-year operator training time and the lack of evidence of effectiveness, its use as a haemodynamic monitor is limited in clinical practice.

### Transoesophageal Doppler Monitoring

Deltex (Deltex Medical, Chichester, UK) developed transoesophageal Doppler monitoring for perioperative monitoring of the descending thoracic aorta [92]. The CW technology is well established as a fluid optimisation methodology with the highest levels of evidence of effectiveness [3•]. However, the method is largely limited to perioperative monitoring as the ultrasound transducer is inserted into the oesophagus and requires sedation. The probes are single-patient use and relatively expensive. Additionally, the ultrasound beam requires frequent re-alignment for accurate monitoring as the peristaltic waves of the oesophagus and respiratory movements move the beam focus relative to the descending thoracic aorta.

### Transcutaneous Doppler Monitoring

**Fig. 7:**
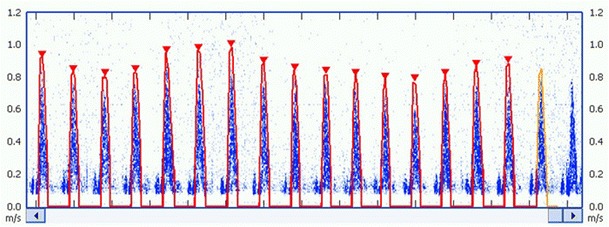
Normal stroke volume respiratory excursion from a CW Doppler flow profile obtained using an USCOM 1A haemodynamic monitor with stroke volume autotracing using FlowTracer®

The USCOM 1A (Uscom Ltd., Sydney, Australia) is a direct CW Doppler SV monitor which can access either the aortic valve from the suprasternal notch, or the pulmonary valve from the parasternal access window [26, 55, 56, 93]. The device is simple to learn and use, requiring the trainee to perform about 30 studies to achieve competance. It generates a real time beat-to-beat SV, using an auto-traced Doppler signal which yields multiple advanced haemodynamic parameters simultaneously. Critchley et al. compared the Cardio-Q and USCOM 1A and concluded that although both showed good agreement, at higher and lower outputs, the USCOM 1A was probably more reliable [94]. The trend screens are designed for simplified operation and accurate SV monitoring over a short time, such as during SV respiratory variability studies to determine fluid responsiveness, or over longer times to assess the effectiveness of CV interventions (Fig. 7).

## Conclusion

Doppler ultrasound has been used since 1980 to measure cardiovascular blood flow, and its applications and protocols for use are now well established. Doppler ultrasound is a sophisticated technology and its application to advanced haemodynamics depends on the training and skill of the operator, and an understanding of the unique physical properties of Doppler ultrasound [91•]. New specialised Doppler ultrasound devices deliver real-time SV measures simply and rapidly, and, combined with central and brachial BP monitoring, provide multi-parametric indices which can provide non-invasive flow-pressure parameters which improve on simple BP monitoring and which may provide novel insights into cardiovascular physiology, pathophysiology and therapy.
